# Mink is an important possible route of introducing viral respiratory infections into the human population

**DOI:** 10.1002/vms3.1177

**Published:** 2023-06-12

**Authors:** Mohammad Hossein Razizadeh

**Affiliations:** ^1^ Department of Virology Faculty of Medicine Iran University of Medical Sciences Tehran Iran

Minks are small carnivorous mammals that belong to the family Mustelidae. Currently, two species of minks are living named American mink (*Neovison vison*) and European mink (*Mustela lutreola*), which the former is more popular for its fur, and due to the high demand for its fur, it has been imported to Europe. Because of their escape from mink farms and deliberate release, there are many colonies of American mink living in different parts of Europe (Bonesi & Palazon, 2007).

Thus far, some viral agents able to infect and cause disease in humans such as Rotavirus C and Hepatitis E virus have been detected in minks (Xie et al., 2018). The severe acute respiratory syndrome coronavirus 2 has caused several outbreaks in farmed minks, leading to the mass cull of them in some countries to control the infection (Koopmans, 2021; Oude Munnink et al., 2021). In recent years, virologic studies have found the infection of minks with viral infection of other animals. Zhao et al. reported a fatal outbreak of Newcastle virus, which infects avian species, among the European minks in China (Zhao et al., 2017). Recently, the outbreak of highly pathogenic avian influenza A (H5N1) virus infection in mink has attracted much attention. Phylogenetic analysis showed the isolated viruses to be 99.8%–100% similar to A/ gull/France/22P015977/2022‐like genotype (Agüero et al., 2023). A study by Sun et al. (2021) reported the presence of antibodies against human (H3N2 and H1N1/pdm) and avian (H7N9, H5N6 and H9N2) influenza A viruses in minks in China was published a year before the recent report. Therefore, it is important to conduct further research on the ability of minks to obtain respiratory viruses and evolve them to fit the mammalian body. A summary of viruses with the ability to cause disease in humans, which were detected in mink, is presented in Table 1.

**TABLE 1:** List of viruses isolated from minks that can infect humans.

Virus	Note
Influenza A virus H3N2 and H1N1/pdm	Besides two human‐infecting strains, minks were also infected with three avian strains (H7N9, H5N6 and H9N2) (Sun et al., 2021)
SARS‐CoV‐2	The receptor used for viral entry is very similar in both human and mink (Fenollar et al., 2021)
Astrovirus	The higher infection rate in the summer indicates a seasonality in infection trends (Xie et al., 2018)
Rotavirus C	An increase in numbers of positive farms has been reported (Xie et al., 2018)
Hepatitis E	Xie et al. (2018)
Aleutian mink disease parvovirus	The virus has been isolated from two mink farmers with vascular disease and microangiopathy (Jepsen et al., 2009)

Identification of various viral families in both mink species in different parts of the world indicates the possible role of this animal to adapt viruses of other classes, especially avians, to mammals and the potential role of minks to cause future outbreaks of viral infection in the human population. Moreover, due to the possibility of transmitting viruses such as SARS‐CoV‐2 by mink to other animals and humans (Fenollar et al., 2021), public healthcare authorities should be alarmed of the potential threat of developing zoonotic diseases, and proper measures to avoid future outbreaks are required to be implemented. Moreover, considering the capability of minks to contract both avian and human Influenza strains, studies on their possible role in the evolution of Influenza viruses are suggested.

## AUTHOR CONTRIBUTIONS

The conceptualization, preparation of the manuscript, and revision were accomplished by Mohammad Hossein Razizadeh.

## CONFLICT OF INTEREST STATEMENT

The author declares no competing interests.

### PEER REVIEW

The peer review history for this article is available at https://publons.com/publon/10.1002/vms3.1177.

## Data Availability

Not applicable.

## References

[vms31177-bib-0001] Bonesi, L. , & Palazon, S. (2007). The American mink in Europe: Status, impacts, and control. Biological Conservation, 134(4), 470–83.

[vms31177-bib-0002] Xie, X‐T. , Macdonald, R. E. , Tapscott, B. , Nagy, E. , & Turner, P. V. (2018). Detection of astrovirus, rotavirus C, and hepatitis E viral RNA in adult and juvenile farmed mink (*Neovison vison*). Frontiers in Veterinary Science, 5, 132. 29974054 10.3389/fvets.2018.00132 PMC6020771

[vms31177-bib-0003] Koopmans, M. (2021). SARS‐CoV‐2 and the human‐animal interface: Outbreaks on mink farms. The Lancet Infectious Diseases, 21(1), 18–9. 33227234 10.1016/S1473-3099(20)30912-9 PMC7832374

[vms31177-bib-0004] Oude Munnink, B. B. , Sikkema, R. S. , Nieuwenhuijse, D. F. , Molenaar, R. J. , Munger, E. , Molenkamp, R. , van der Spek, A. , Tolsma, P. , Rietveld, A. , Brouwer, M. , Bouwmeester‐Vincken, N. , Harders, F. , Hakze‐van der Honing, R. , Wegdam‐Blans, M. C. A. , Bouwstra, R. J. , GeurtsvanKessel, C. , van der Eijk, A. A. , Velkers, F. C. , Smit, L. A. M. , … Koopmans, M. P. G. (2021). Transmission of SARS‐CoV‐2 on mink farms between humans and mink and back to humans. Science, 371(6525), 172–7. 33172935 10.1126/science.abe5901 PMC7857398

[vms31177-bib-0005] Zhao, P. , Sun, L. , Sun, X. , Li, S. , Zhang, W. , Pulscher, L. A. , & Xing, M. (2017). Newcastle disease virus from domestic mink, China, 2014. Veterinary Microbiology, 198, 104–7. 28061999 10.1016/j.vetmic.2016.12.003

[vms31177-bib-0006] Agüero, M. , Monne, I. , Sánchez, A. , Zecchin, B. , Fusaro, A. , Ruano, M. J. , Del Valle Arrojo, M. , Fernández‐Antonio, R. , Souto, A. M. , Tordable, P. , Cañás, J. , Bonfante, F. , Giussani, E. , Terregino, C. , & Orejas, J. J. (2023). Highly pathogenic avian influenza A (H5N1) virus infection in farmed minks, Spain, October 2022. Eurosurveillance, 28(3), 2300001. 36695488 10.2807/1560-7917.ES.2023.28.3.2300001 PMC9853945

[vms31177-bib-0007] Sun, H. , Li, F. , Liu, Q. , Du, J. , Liu, L. , Sun, H. , Liu, J. , Zhang, X. , Yang, J. , Duan, Y. , Bi, Y. , Pu, J. , Sun, Y. , Tong, Q. , Wang, Y. , Du, X. , Shu, Y. , Chang, K. C. , & Liu, J. (2021). Mink is a highly susceptible host species to circulating human and avian influenza viruses. Emerging Microbes & Infections, 10(1), 472–80. 33657971 10.1080/22221751.2021.1899058 PMC7993395

[vms31177-bib-0008] Fenollar, F. , Mediannikov, O. , Maurin, M. , Devaux, C. , Colson, P. , Levasseur, A. , & Raoult, D. (2021). Mink, SARS‐CoV‐2, and the human‐animal interface. Frontiers in Microbiology, 12, 663815. 33868218 10.3389/fmicb.2021.663815 PMC8047314

[vms31177-bib-0009] Jepsen, J. R. , Amore, F. , Baandrup, U. , Clausen, M. R. , Gottschalck, E. , & Aasted, B. (2009). Aleutian mink disease virus and humans. Emerging Infectious Diseases, 15(12), 2040. 19961696 10.3201/eid1512.090514 PMC3044528

